# Towards Soft Wearable Strain Sensors for Muscle Activity Monitoring

**DOI:** 10.1109/TNSRE.2022.3196501

**Published:** 2022-08-11

**Authors:** Jonathan T. Alvarez, Lucas F. Gerez, Oluwaseun A. Araromi, Jessica G. Hunter, Dabin K. Choe, Christopher J. Payne, Robert J. Wood, Conor J. Walsh

**Affiliations:** John A. Paulson School of Engineering and Applied Sciences, Harvard University, Cambridge, MA 02138 USA; John A. Paulson School of Engineering and Applied Sciences, Harvard University, Cambridge, MA 02138 USA; John A. Paulson School of Engineering and Applied Sciences, Harvard University, Cambridge, MA 02138 USA; John A. Paulson School of Engineering and Applied Sciences, Harvard University, Cambridge, MA 02138 USA.; John A. Paulson School of Engineering and Applied Sciences, Harvard University, Cambridge, MA 02138 USA; John A. Paulson School of Engineering and Applied Sciences, Harvard University, Cambridge, MA 02138 USA. He is now with Viam, Inc., New York City, NY 10023 USA.; John A. Paulson School of Engineering and Applied Sciences, Harvard University, Cambridge, MA 02138 USA; John A. Paulson School of Engineering and Applied Sciences, Harvard University, Cambridge, MA 02138 USA

**Keywords:** Muscle activity monitoring, wearable sensors, muscle deformation sensors, soft strain sensors, biomedical transducers, biomedical monitoring

## Abstract

The force-generating capacity of skeletal muscle is an important metric in the evaluation and diagnosis of musculoskeletal health. Measuring changes in muscle force exertion is essential for tracking the progress of athletes during training, for evaluating patients’ recovery after muscle injury, and also for assisting the diagnosis of conditions such as muscular dystrophy, multiple sclerosis, or Parkinson’s disease. Traditional hardware for strength evaluation requires technical training for operation, generates discrete time points for muscle assessment, and is implemented in controlled settings. The ability to continuously monitor muscle force without restricting the range of motion or adapting the exercise protocol to suit specific hardware would allow for a richer dataset that can help unlock critical features of muscle health and strength evaluation. In this paper, we employ wearable, ultra-sensitive soft strain sensors for tracking changes in muscle deformation during contractions. We demonstrate the sensors’ sensitivity to isometric contractions, as well as the sensors’ capacity to track changes in peak torque over the course of an isokinetic fatiguing protocol for the knee extensors. The wearable soft system was able to efficiently estimate peak joint torque reduction caused by muscle fatigue (mean NRMSE = 0.15±0.03).

## I. INTRODUCTION

The force output capacity of skeletal muscle encodes rich information about several aspects of human health. Fluctuations in the force output of skeletal muscle (muscle strength) are indicative of lower-grade injury, for example, exercise-induced muscle damage following intense physical activity [1], [2]. Therefore, the ability to measure the force-generating capacity of muscles over a period of time is useful for individuals seeking to improve their training regimens and reduce the risk of overtraining and injury [3], [4]. Additionally, a decline in the force production capacity of skeletal muscle precedes several medical conditions, such as muscular dystrophy, multiple sclerosis, and Parkinson’s disease [5]. Consequently, the assessment of muscle force generation is also important for clinicians evaluating the overall health or recovery of their patients.

Monitoring muscle force output over a period of time could enable safer and more efficient clinical rehabilitation and athletic training protocols. Longitudinal collection of data at finer time points is less susceptible to within- or across-day fluctuations in force generation. However, long-term evaluation (over the period of hours or days) of muscle force output in real-world contexts is currently challenging, due to a lack of robust techniques capable of taking such measurements outside of the lab or clinical settings [6].

Traditional clinic-based muscle strength assessment methods include manual muscle testing (MMT) and isokinetic dynamometry. MMT is a clinician-administered exam, which scores a patient’s muscle strength between zero and five, depending on their ability to both move through a full range of motion and resist applied pressure. The MMT is easily administered with no associated cost, but it is a discrete and subjective measurement subject to high variability [7]. Isokinetic dynamometry is the current gold-standard for evaluating muscle strength. Isokinetic dynamometry measures joint torque during static or constant-velocity movements [8]. Isokinetic dynamometers also measure rotational speed, position, and direction, allowing for precise and controlled measurements of muscle force acting about a joint. However, these machines are cost-prohibitive for most hospitals and clinics. Also, factors such as age, weight, or severity of injury can make evaluation with an isokinetic dynamometer impractical for certain patient populations. Ultimately, both the MMT and isokinetic dynamometry can only be administered in controlled settings and at discrete time points (typically weeks, months, or years) [7]–[10].

Researchers and clinicians have become interested in wearable systems over the last decades because these systems have the potential to continuously monitor muscle activity, in addition to being lightweight, low-profile, and affordable. [11]–[13]. Sensing technologies, such as surface electromyography (sEMG) [14]–[16], mechanomyography (MMG) [17]–[19], and force myography (FMG) [20], [21], have all been proposed as possible solutions to this challenge of ubiquitous monitoring of muscle force, each with varying levels of success.

sEMG is considered the gold-standard for wearable sensors measuring muscle activity, given its commercial adoption and wide range of applications. sEMG uses electrodes to measure the bulk electrical potential within muscle fibers, and this signal is indicative of the activation level of muscle [14]. However, as sEMG is primarily a measure of electrical activity within a muscle, its relationship to the mechanical output of muscle remains elusive [15], [22]. Crosstalk from adjacent co-contracting muscles can also contaminate the signal from the muscle of interest, complicating interpretation [16]. MMG, considered the mechanical counterpart to sEMG, measures the lateral oscillations of contracting muscle fibers with microphones or accelerometers, and has steadily gained in popularity over the past years. MMG does not require electrodes for signal transduction and is unaffected by inherent electrical noise, which affects sEMG [19]. The main challenge for MMG is measurement during dynamic contractions, as motion artifacts can severely deteriorate signal quality [17], [18]. Lastly, FMG belongs to a broader category of sensing techniques attempting to measure the force generated by a muscle externally by quantifying how muscle geometry changes. FMG typically uses a band of pressure sensors wrapped around the limb of interest to measure radially-directed pressure as the muscle bulges outwards. However, by radially-constraining all sensors in a band wrapped around the limb of interest, any muscle that bulges under the band will affect the signal of all sensors. The resulting mechanical coupling between the sensors makes interpretation of the FMG output challenging. Sensitivity is another consideration in FMG systems, as most off-the-shelf pressure or force sensors are not able to detect small motions of the underlying muscle, thus FMG bands typically need to be carefully pretensioned to compensate for a lack of sensitivity [20].

Current research on sEMG, MMG, and FMG has focused on gathering more data with additional sensors (e.g. sEMG arrays), combining multiple sensing techniques (e.g. sEMG and MMG), or using algorithmic advances in post-processing methods (e.g. data-driven machine learning models) to compensate for limitations in the respective sensing technologies. Some of these limitations are inherent to the sensing technology, such as crosstalk in sEMG signals, motion artifacts in MMG sensors, or sensor preloading in FMG, and remain a challenge.

Recently, researchers have investigated the relationship between muscle morphological changes and joint torque during contractions [23]–[25]. In these studies, ultrasound is used to quantify the deformation of skeletal muscle (e.g. changes in width and thickness) during isometric contractions. These architectural changes have been shown to correlate with joint torque. Researchers have also demonstrated that during sustained, fatiguing, isometric contractions, the deformation of contracting muscles varies over time, alongside corresponding reductions in measured joint torque [26]. While ultrasound systems are unaffected by crosstalk and motion artifacts, they cannot be easily integrated into wearable hardware and therefore are mainly limited to isometric studies. Being able to measure muscle deformation with a completely wearable and unrestrictive system could provide researchers and clinicians with a new approach of monitoring muscle activity.

In this paper, we propose a method of estimating static and dynamic changes in muscle force using soft strain sensors that can non-invasively measure muscle deformation. The soft strain sensor [27] is low-profile, robust, and hypersensitive to underlying motion, thus being a candidate technology to address the aforementioned challenges. We hypothesize that adhering the soft strain sensors directly to the surface of the skin above a muscle would allow the sensor to capture changes in muscle deformation which would correlate with muscle force. As a proof of concept, we ran an experiment with eight healthy participants to track muscle force during static and dynamic contractions of the quadriceps muscle and compared the results with data collected from a dynamometer as ground truth.

## II. METHODS

### A. Soft Strain Sensors

The soft strain sensors used in this study were based on the principle of strain-mediated contact in anisotropically resistive structures (SCARS) reported in [27]. SCARS sensors are compliant, resilient, and ultra-sensitive strain sensors, whose primary transduction mechanisms rely on changes in Ohmic contact between adjacent prestrained carbon fiber meanders. As the sensor is strained, the meanders separate, increasing overall resistance. SCARS sensors are low-profile, resilient, washable, and highly sensitive strain sensors, and are therefore well-suited for apparel integration.

Sensor fabrication followed methods described in [27]. Each sensor consisted of symmetric layers of pressure-sensitive silicone adhesive and prestrained thermoplastic polyurethane (TPU) sandwiching a carbon fibre polymer composite (CFPC) meander. Electrical connections to the sensor were made mechanically by securing electrical wire into terminating slots cut into the CFPC meander. A fixed current was passed through the meander, using an LM334 current source regulator (Texas Instruments, USA), and the voltage drop across the resistive CFPC meander was read with a PowerLab 8/35 external data acquisition unit (AD Instruments, New Zealand). A four-point voltage measurement was used to eliminate the effects of contact resistance. Final dimensions of the sensor were a 10 mm × 10 mm CFPC meander encapsulated in a 11 mm wide × 55 mm long TPU layer.

### B. Principle of Operation

**Fig. 1.**
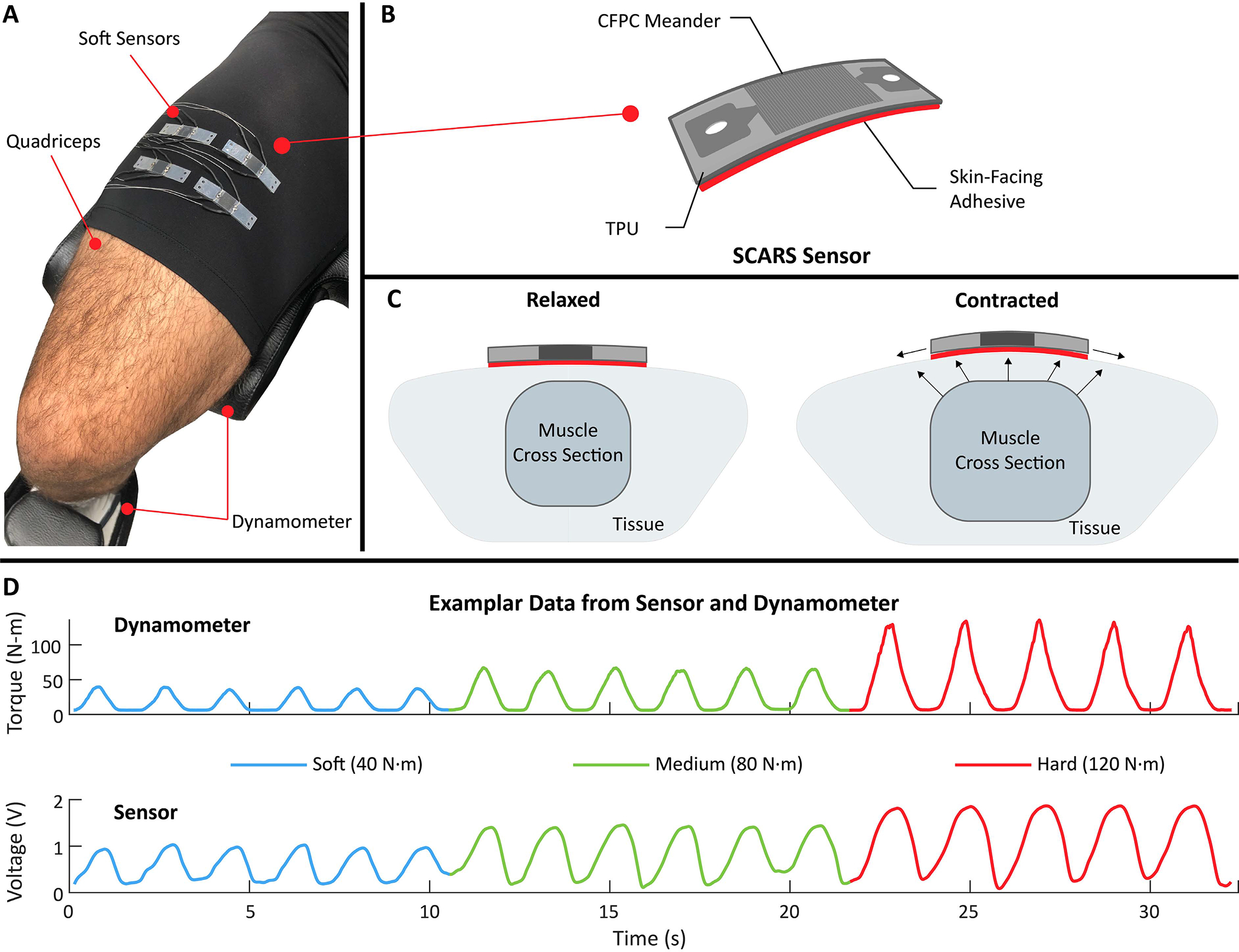
Overview of the study, employing soft, ultra-sensitive strain sensors for muscle activity monitoring. A) The soft sensors are placed over the quadriceps to detect mechanical changes of the muscle during contraction (muscle deformation). A dynamometer was used to provide a ground truth measure of joint angle and torque generated by the muscle of interest. B) The soft sensor is composed of a carbon fibre polymer composite (CFPC) meander encapsulated by prestrained thermoplastic polyurethane (TPU) layers. The sensor is attached perpendicular to the longitudinal axis of the muscle using a double-sided adhesive layer. C) During a contraction, the muscle deforms and distends the skin. As a result, the sensor is strained and its resistance increases. D) The voltage reading from the soft sensor is proportional to the force applied by the muscle. The example from one participant shows the raw sensor voltage and the dynamometer torque for three intensities of contractions of the quadriceps (soft, medium, and hard), ranging from 40 N·m to 120 N·m.

**Fig. 2.**
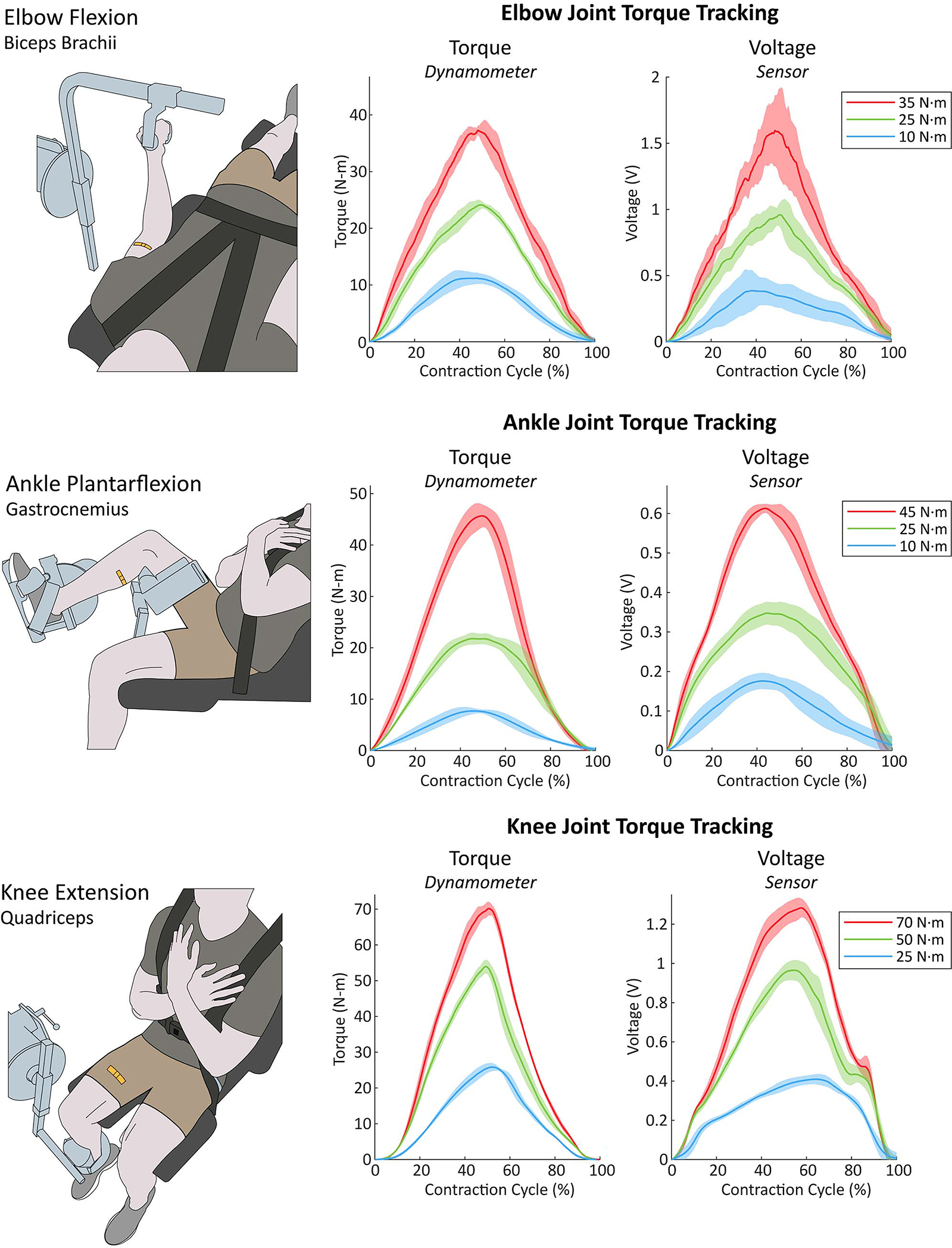
Demonstration of sensor response to 90° isometric contractions for three different muscle groups. The plots show the sensor and dynamometer responses for the isometric contractions, at three different torque values. The shaded areas represent the standard deviation of five contractions at each torque level. All data low pass filtered with a cutoff of 5 Hz.

The sensors were placed on top of the muscle of interest using double-sided adhesive (Vapon Topstick, Vapon, USA), as shown in Fig. 1A. The sensor can measure muscle deformation when adhered directly to the skin or directly to a think layer of textile. By adhering the entire length of the sensor directly above the muscle of interest (Fig. 1B), we were able to detect muscle deformation during a contraction as a change in resistance of the soft sensor (Fig. 1C and Fig. 1D). The muscle contraction causes the muscle belly to deform and distend the skin while shortening [28]. Therefore, by adhering the sensor perpendicular to the longitudinal (shortening) axis of the muscle, the sensor will strain axially during a muscle contraction, as the muscle shortens and deforms in thickness and width. The relationship between the sensor response and muscle force with a set of varying force, isometric contractions can be seen in Fig. 2 for three muscle groups (quadriceps, biceps brachii, and gastrocnemius).

### C. Isokinetic Dynamometer

An isokinetic dynamometer (HUMAC Norm, CSMi Solutions, USA) was used as a ground truth measure of joint torque to compare to the sensors. Joint torque was used as a proxy for muscle force because it is not possible to directly and non-invasively measure forces on individual muscles. There are two main modes on a dynamometer that are used to evaluate muscle function: isometric and isokinetic. An isometric contraction is a fixed joint angle contraction, where there is an increase in muscle force with no corresponding change in muscle tendon unit length. An isokinetic contraction is a constant velocity contraction through a fixed range of motion, where both muscle length and tension are allowed to vary. An isokinetic contraction can either be concentric or eccentric depending on if the muscle shortens or lengthens, respectively, during the contraction.

### D. Experimental Protocol

The experimental protocol assessed: (1) the correlation between sensor response and knee joint torque during isometric contractions, (2) the sensor’s ability to track reductions in peak knee joint torque caused by fatigue during sequential isokinetic concentric contractions, and (3) how sensor placement affects the relationship between sensor response and joint torque.

Eight healthy participants (seven males, one female; mean±SD: age: 27.8±3.8 years; height: 1.80±0.09 m; mass: 76.1±12.4 kg) were recruited to participate in the experimental protocol. All participants self-reported as moderate to highly active individuals and the right leg was evaluated, regardless of dominance. All participants gave written consent prior to participating and the protocols were approved by the Harvard Medical School Institutional Review Board under protocol IRB17-1201.

Upon arrival to the laboratory participants were given a pair of tight fitting compression shorts to wear for the duration of the study. The participants were led through a warm-up consisting of static and dynamic lower limb stretches, and then instructed to mount the dynamometer. Following a set procedure, the dynamometer and chair were adjusted to the participants anatomy, ensuring the center of the knee joint (lateral epicondyle of the femur) was aligned with the dynamometer’s axis of rotation. Next, participants were secured to the chair with straps across the chest to minimize compensatory trunk movements. The resistance pad on the lever arm of the dynamometer was secured to the distal two-thirds of the participant’s tibia. A calibration procedure was performed to establish the participant’s range of motion (ROM), which set both soft and hard stops at either end of their ROM for safety. Finally, participants were asked to perform a set of submaximal isometric and isokinetic knee contractions to familiarize themselves with the ensuing protocol.

Four soft sensors were placed on the participants’ quadriceps in a 2 × 2 grid pattern, centered on the rectus femoris and located midway between the greater trochanter and the lateral epicondyle of the femur. All sensors were adhered directly to compression shorts using double-sided adhesive. The compression shorts were secured to the participant with additional tape to prevent any translation or rotation of the compression shorts during the testing protocol. After initial placement, participants were instructed to contract their quadriceps isometrically, and the response of the sensors was noted by the researchers. Positional adjustments of the sensors were performed until it was observed that the muscle underneath each sensor deformed monotonically (strictly in one direction) during the full range of force production, without saturation of the sensor.

**Fig. 3.**
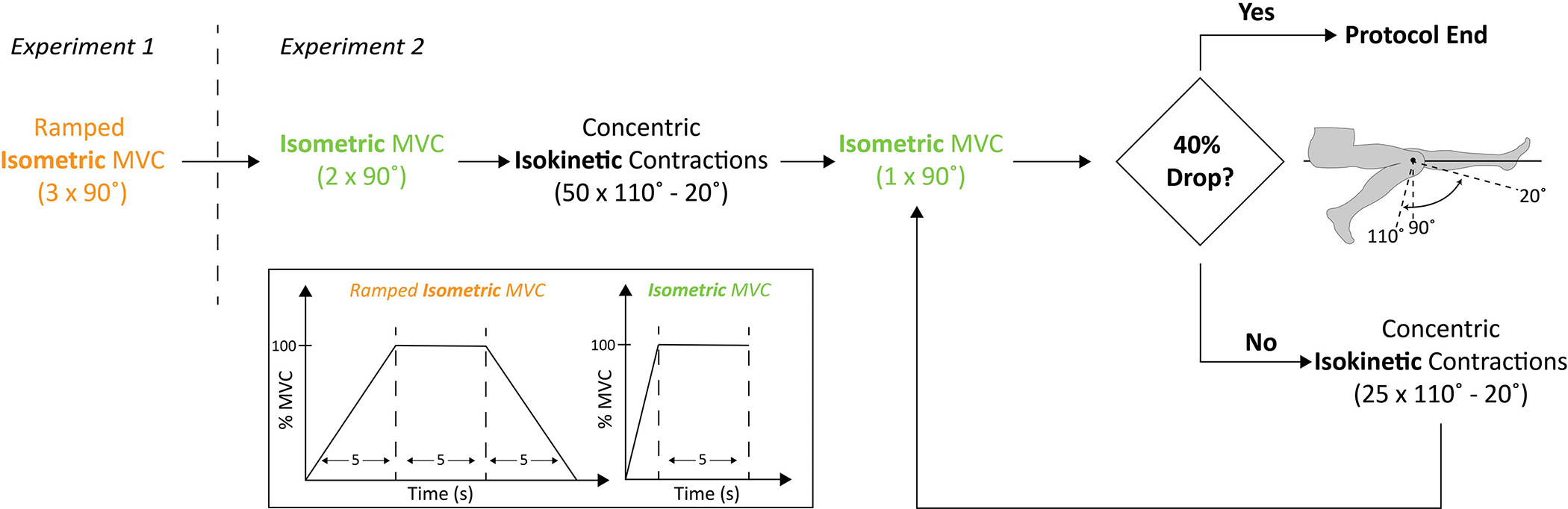
The main testing protocol was split into two experiments. *Experiment 1* was composed of a set of three ramped isometric maximum voluntary contractions (MVC) at a knee angle of 90°. In *experiment 2* the participants were first instructed to perform two baseline isometric MVCs at a knee angle of 90°. Unlike the first set of ramped contractions, there was no ramping involved in these contractions, and participants were simply instructed to apply and hold max effort for a total of five seconds. Immediately after the two isometric contractions, participants performed maximal effort isokinetic contractions of the quadriceps. These contractions ranged 90° (from 110° to 20°) at an angular velocity of 30°/s. After 50 contractions, participants were instructed to perform one isometric MVC at 90°. If the peak torque had decreased by 40% relative to the baseline, the protocol was ended. If the participant’s peak torque had not decreased by 40%, additional sets of 25 maximal isokinetic contractions were performed until the 40% deficit in peak isometric torque was reached.

The exercise protocol consisted of two experiments (Fig. 3): sensor calibration (*experiment 1*) and the fatiguing protocol (*experiment 2*). Additionally, a third experiment was performed on a single participant to evaluate the sensor’s sensitivity to placement (*experiment 3*).

#### 1) Experiment 1 (Sensor Calibration):

Participants were asked to perform a series of three ramped isometric maximum voluntary contractions (MVCs) at a knee angle of 90°. Each ramped MVC consisted of a five-second ramp from 0 to 100% effort, a five-second hold at 100% effort, and a five-second ramp down to rest. Each MVC was separated by 60 seconds for recovery (Fig. 3).

#### 2) Experiment 2 (Fatiguing Protocol):

The fatiguing protocol consisted of a set of consecutive maximum effort isokinetic concentric contractions (Fig. 3) designed to cause a 40% reduction in peak isometric torque (adapted from [29]).

Participants were first instructed to perform two baseline isometric MVCs at a knee angle of 90°, separated by 30 seconds. Following the two baseline isometric contractions, participants performed sequential repetitions of maximal effort isokinetic concentric contractions of the quadriceps, from 110° to 20° of flexion at an angular velocity of 30°/s. After completion of each concentric contraction, the participants were instructed to lower their legs with minimal effort back to 110° at an angular velocity of 60°/s. Following the completion of 50 isokinetic concentric contractions, participants performed one isometric MVC at a knee angle of 90°. If their peak torque decreased by 40% relative to their baseline measure, the protocol was ended. However, if the participant’s peak torque decreased by less than 40%, they performed additional sets of 25 maximal isokinetic contractions until the 40% deficit in peak isometric torque was reached.

As an additional data source, three bipolar surface EMG electrodes (TELEmyo 2400 G2, Noraxon, USA) were placed on the vastus lateralis, rectus femoris, and vastus medialis muscles to measure electrical activity during the protocol. Electrodes were placed following SENIAM recommendations for each of the target muscles [30]. sEMG data was not collected for participant 4 due to technical issues with the sEMG system during the study.

#### 3) Experiment 3 (Sensor Sensitivity to Placement):

On a single participant, a 5 × 5 grid (10 mm × 10 mm cells) was drawn on the quadriceps centered at a location where it was observed that the muscle underneath the sensor deformed monotonically during the full range of force production, without saturation of the sensor. Next, the sensor’s response to three 100 N·m. isometric contractions was sequentially evaluated at each of the 25 locations.

### E. Data Processing and Outcome Metrics

Data was logged simultaneously at 1kHz with the data acquisition unit, including two analog outputs from the dynamometer corresponding to torque and position, as well as the four sensor inputs. The dynamometer torque and sensor data were low-pass filtered using a zero-phase Butterworth lowpass filter with a cutoff frequency of 5 Hz. For all experiments, dynamometer torque and sensor data were normalized to their maximum values.

#### 1) Experiment 1 (Sensor Calibration):

For *experiment 1*, the rising portion (defined as onset of contraction to peak torque) of each isometric contraction was segmented for the dynamometer and sensor data. The coefficient of determination (*r*^2^) between each sensor and torque was calculated for each participant (subject-specific calibration). Using the sensor with the highest *r*^2^ score per participant, a single cubic fit (group calibration) was created, defining a general relationship between normalized torque and sensor response on the quadriceps. Next, the mean *r*^2^ and the mean normalized root mean square error (NRMSE) between the sensor-estimated and dynamometer-measured torque was calculated for the three isometric contractions for the subject-specific and group calibrations across all participants.

#### 2) Experiment 2 (Fatiguing Protocol):

For *experiment 2*, the peak torque and peak sensor voltage per isokinetic contraction were calculated. Next, the generalized cubic fit (group calibration) calculated in *experiment 1*, describing sensor voltage correlation to joint torque, was applied to the peak sensor data. Thus, for all participants, the peak dynamometer-measured torque and peak sensor-estimated torque were calculated, per contraction, and used for analysis. The sensor peak variance was then calculated using Principal Component Analysis (PCA), an unsupervised dimensionality reduction technique. The sensor with the highest variance (highest first principal component, PC1) per participant was selected for analysis.

To analyze the level of muscular fatigue, it is common to compare the force generation capacity of the muscle at the beginning and end of exercise protocols [31]–[33]. Therefore, in *experiment 2*, we compared the sensor-estimated torque to the dynamometer-measured torque at the beginning (first five contractions) and at the end (last five contractions) of the fatiguing protocol. Additionally, we calculated the within-subject correlations for repeated measures to analyze associations between the peak sensor and torque readings, while accounting for variation between participants (e.g. sensor placement, participant’s muscle structure, or participant’s resistance to fatigue). This technique is known as a Repeated Measures Correlation, and is similar to a null multilevel model of varying intercept while maintaining a common slope for all participants. The analysis was conducted in R programming language using the *rmcorr* package [34].

Raw sEMG signals from *experiment 2* were filtered with a fourth-order band-pass Butterworth filter with cutoff frequencies of 20–400 Hz to remove electrical noise and biological artifacts. The filtered sEMG data was then processed to calculate the mean median frequency (MDF) across all three muscles, per contraction.

#### 3) Experiment 3 (Sensor Sensitivity to Placement):

For *experiment 3*, the rising portion of each isometric contraction was segmented for the dynamometer and sensor data, at each of the 25 locations (5 × 5 grid) drawn on the participant’s quadriceps. Next, the generalized cubic fit (group calibration) defined in *experiment 1* was applied to the sensor data and the mean *r*^2^ across the three 100 N·m isometric contractions was calculated at each location.

## III. EXPERIMENTAL RESULTS

### A. Experiment 1 (Sensor Calibration)

**Fig. 4.**
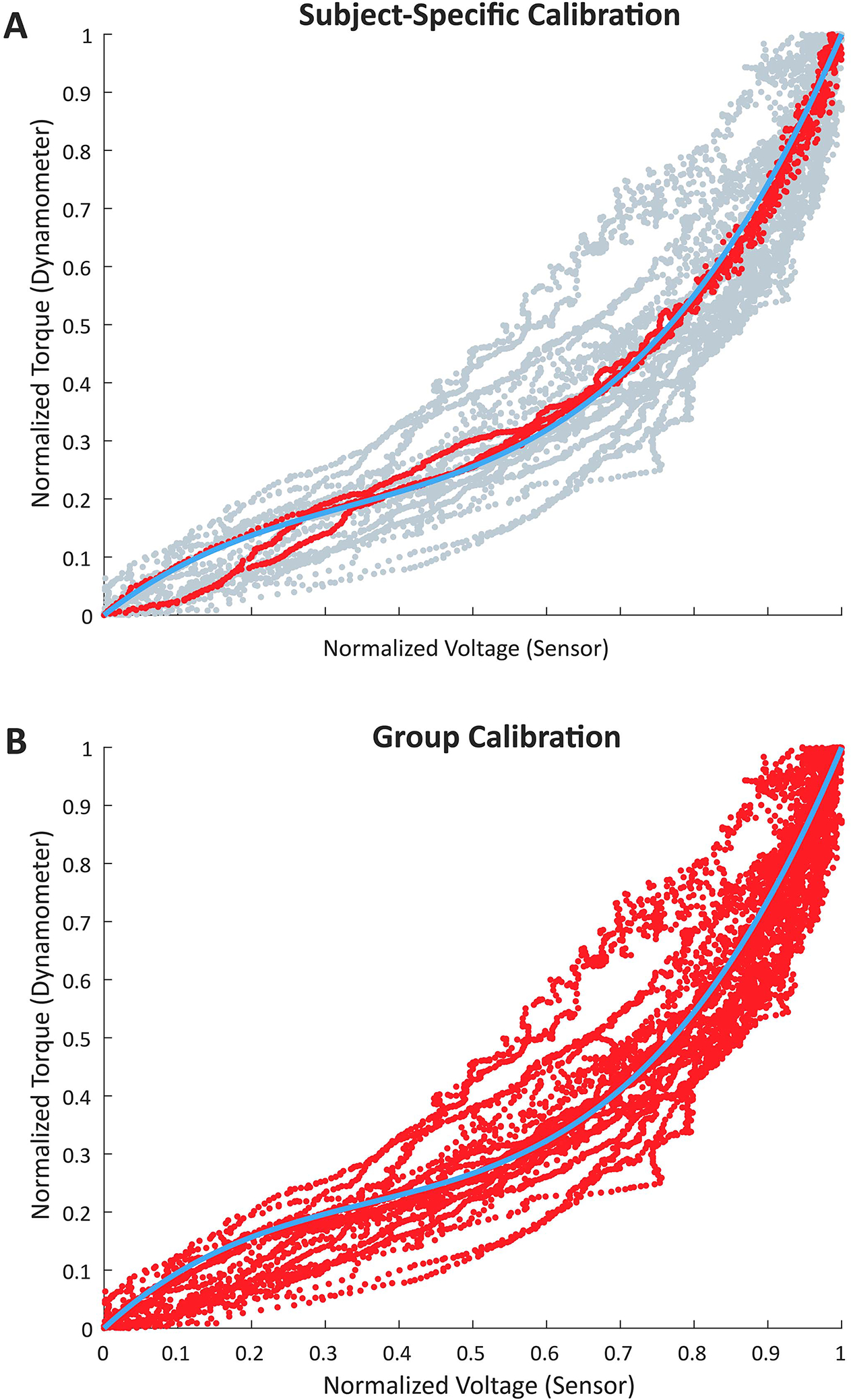
Least squares estimation of the relationship between the sensor and joint torque during isometric contractions. A) Example sensor and torque relationship for a single participant, with overlaid cubic fit (blue). B) Group level data across all eight participants, with group fit overlaid in blue.

The relationship between the sensor signal and joint torque during *experiment 1* is highlighted in Fig. 4A for a single participant and in Fig. 4B for all eight participants.

The mean NRMSE between the sensor-estimated and ground truth torque for the subject-specific calibration was 0.07±0.03 with an *r*^2^ of 0.93±0.05. The NRMSE between the sensor-estimated and ground truth torque for the group calibration was 0.09, with an *r*^2^ across all eight participants of 0.90.

### B. Experiment 2 (Fatiguing Protocol)

**Fig. 5.**
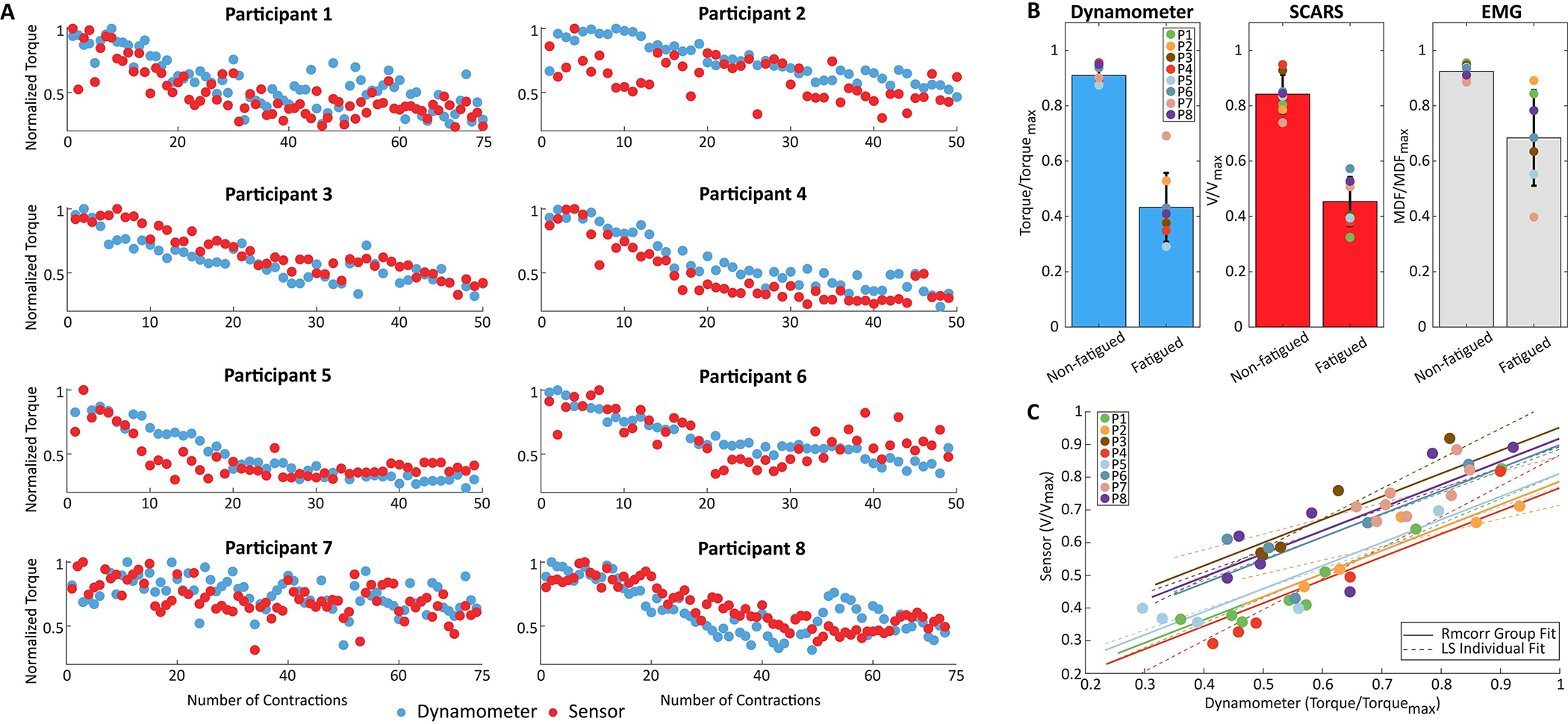
Summary of the results for the fatiguing protocol on the quadriceps for eight participants. A) Normalized peak sensor and dynamometer values plotted for all contractions during the isokinetic fatiguing protocol on the quadriceps. The results show similar torque and sensor output reduction profiles throughout the protocol for the participants (mean NRMSE = 0.15±0.03). B) Mean normalized values of the first five contractions (Non-fatigued) and last five contractions (Fatigued) for ground-truth torque, sensor-estimated torque, and sEMG median frequency. The results demonstrate that the soft strain sensors were able to capture similar magnitude reductions in joint torque over the course of the fatiguing protocol as compared to the dynamometer and sEMG system; mean signal amplitude reduction of 52.4±13.8% for the dynamometer, 46.1±11.4% for the soft strain sensors, and 26.0±18.8% for the sEMG system across all participants. C) Repeated measures correlations between the normalized sensor and dynamometer outputs. The solid lines represent the intra-individual correlation fit, the dashed lines represent the least-squares linear fit for each subject, with each colored dot corresponding to an average of ten contractions during the fatiguing protocol. The results show a strong, positive correlation between the sensor and dynamometer values (*r*_*rm*_ = 0.73, p<0.001).

The sensor-estimated and dynamometer-measured peak torque, per contraction, for all eight participants is shown in Fig. 5A. Five participants required only 50 contractions before reaching the 40% threshold. Three participants required an additional set of 25 contractions. The mean NRMSE between the sensor-estimated and dynamometer-measured peak torque was 0.15±0.03.

Fig. 5B compares the calibrated sensor signal amplitude to the dynamometer torque at the start and end of the fatiguing protocol. The results show that the soft strain sensors were able to capture muscle force reductions comparable to the dynamometer and the sEMG system; the mean signal amplitude reduced by 52.4±13.8% for the dynamometer, 46.1±11.4% for the soft strain sensors, and 26.0±18.8% for the sEMG system. The repeated measures correlation across participants for the peak torque and sensor values during the fatiguing protocol are shown in Fig. 5C. The results show a strong, positive correlation between the sensor and dynamometer values (*r*_*rm*_ = 0.73, p<0.001), despite differences in body type, muscle anatomy, and strength between participants.

### C. Experiment 3 (Sensor Sensitivity to Placement)

**Fig. 6.**
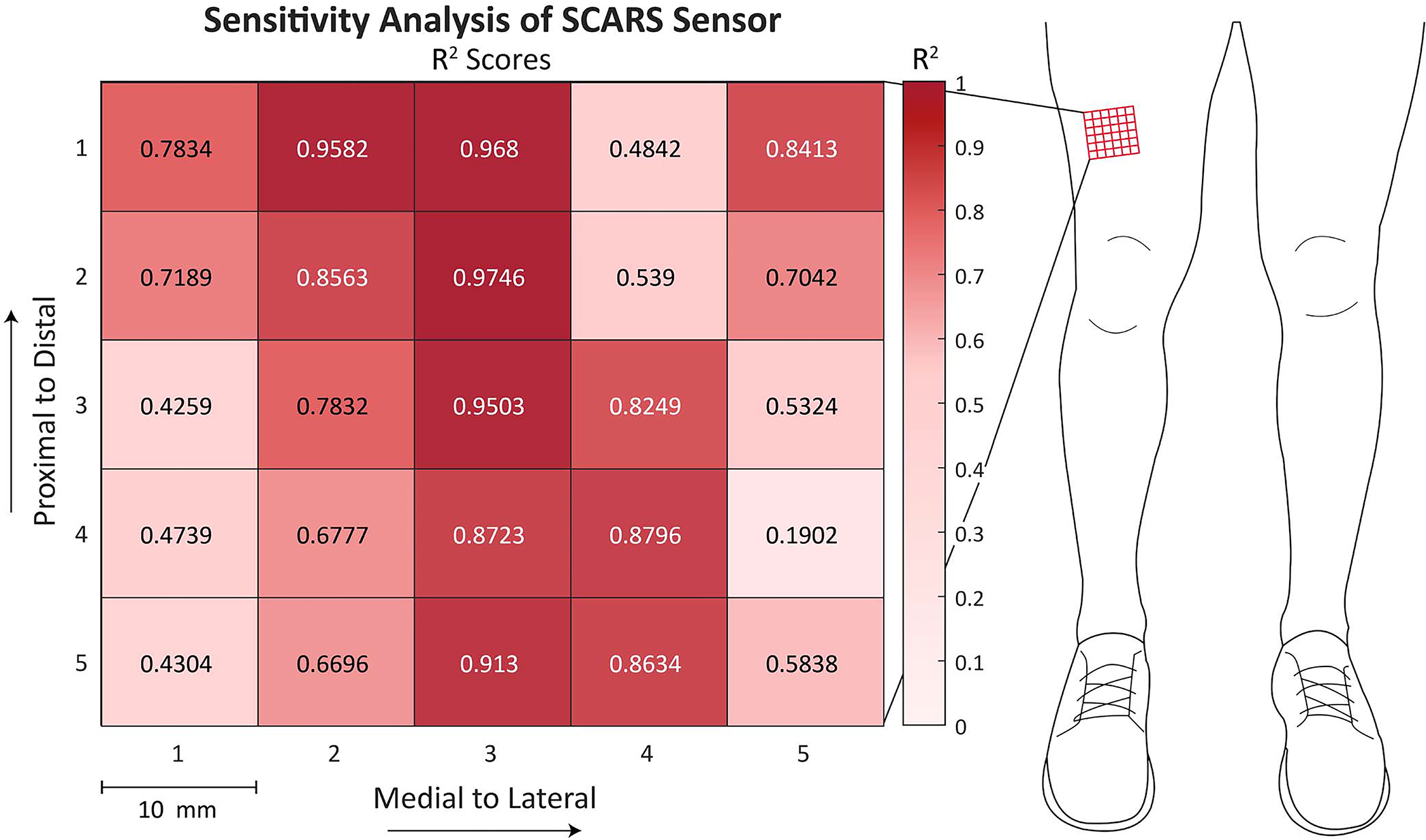
One limitation of the current system is the sensor’s sensitivity to placement. The heat map above shows the *r*^2^ value across three 100 N·m isometric contractions at various locations on the quadriceps of a single participant. A single sensor was placed on 25 different locations on the quadriceps, separated by 10 mm. At each location, three isometric contractions at a knee angle of 90° were performed. For each location, the rising portion of the three contractions was segmented and the global fit defined in *experiment 1* was applied. The *r*^2^ for this cubic fit between sensor and torque for a given location is displayed in the figure above.

Fig. 6 depicts the mean *r*^2^ score between sensor-estimated and dynamometer-measuredtorque for three isometric contractions at 25 different locations on the quadriceps. The maximum *r*^2^ was 0.97 at a position directly distal of the candidate location identified at the start of *experiment 3*. The minimum *r*^2^ was 0.19 at a position proximal and lateral to the starting candidate location. Fig. 6 shows that shifting the sensor by 10 mm can decrease the *r*^2^ by 0.68.

## IV. DISCUSSION AND CONCLUSION

In this paper, we presented a method capable of tracking changes in muscle force output with soft strain sensors by non-invasively measuring muscle deformation. This work aims at advancing sensing capabilities for evaluation of muscle force output towards real-world applications. The ability to monitor muscle force output can offer immense benefits to clinicians or researchers who are looking to track how patients respond to treatment and rehabilitation, or athletes who wish to track and improve their performance during exercise.

As hypothesized, we demonstrated that the adhesion of a soft strain sensor directly above a muscle of interest allows the sensor to capture changes in muscle deformation which correlate with muscle force output. We investigated the feasibility of the proposed method with a study on eight participants during static, isometric contractions and dynamic, fatigue-inducing isokinetic contractions on the quadriceps. We were able to track joint torque on the quadriceps during isometric contractions with an NRMSE of 0.09 as compared to the ground truth torque measured by the dynamometer. Furthermore, we demonstrated how the sensors could be used to track peak torque reductions over the course of a fatiguing protocol consisting of sequential isokinetic contractions of the quadriceps with a mean NRMSE of 0.15±0.03 as compared to the dynamometer. We also saw similar levels of sensor signal amplitude reduction at the end of the protocol compared to the ground truth (46.1±11.4% for the soft sensors and 52.4±13.8% for the dynamometer).

The error between sensor-estimated and dynamometer-measured joint torque during the second experiment can likely be attributed to two main factors. First, in this work we are estimating global joint torque (the result of multiple muscles acting in synergy) with information about muscle deformation at a single muscle. Future work will be needed to understand how information about individual muscle contributions to joint torque in dynamic scenarios can be used to isolate and minimize the effect of these errors on sensor-estimated joint torque. For example, we could explore the impact on estimated joint torque accuracy by adding additional sensors to other contributing muscles to knee joint torque (e.g. vastus medialis and lateralis) and verify their individual contributions to the change in accuracy. The second likely source of error are compensatory movements during fatigue testing, which are a common issue during human-subjects experiments on dynamometers [35], [36]. As hip flexion was not able to be well constrained (due to possible interference with sensors), this may have led to compensatory motion (in order to try and maintain torque) and thus contaminated the overall accuracy of sensor-estimated torque.

While difficult to compare directly to examples in literature, given differences in experimental design, processing techniques, and number of sensors, the proposed sensing system has performed comparably to sEMG, MMG, and FMG. These comparisons are limited to isometric protocols as most studies on wearable sensors do not focus on muscle force estimation during dynamic, isokinetic contractions due to signal-to-noise limitations from motion artifacts or challenges resulting from the onset of fatigue [37], [38]. For example, the relationship between sEMG and MMG signals with muscle force becomes increasingly non-linear as the muscle fatigues [39]. Concerning static, isometric contractions, Staudenmann *et al.* reported an NRMSE of 0.094 between estimated force and sEMG during an 80% isometric MVC of the elbow extensors using an array of 130 monopolar sEMG electrodes [40]. Similarly, Youn *et al.* noted an NRMSE of 0.13 using MMG to estimate isometric elbow flexion force using a feedforward neural network [41]. Castellini *et al.* used a band of 10 force-sensitive resistors to predict finger forces with a cited NRMSE between measured and estimated force ranging from 0.05 to 0.14 for three different machine learning methods [42].

In this work, we designed experiments and processed data in an effort to highlight the practicality of our deformation-based approach and demonstrate its potential for applications outside of the lab or clinic. For example, throughout all experiments the soft sensors were placed over a pair of compression shorts to demonstrate that the sensors could be integrated into apparel without requiring direct contact with the skin. Additionally, a group calibration was used for data processing, instead of a participant-specific calibration, increasing the NRMSE by 0.02 for a more generalizable implementation. Finally, when processing the sensor data from *experiment 2* the isometric calibration was applied, rather than creating a separate calibration for the isokinetic trials, illustrating the generalizability of the deformation-torque relationship. These choices were made to move us closer towards the long-term goal of using deformation-based sensing for longitudinal evaluation and monitoring of muscle force output in the real-world.

Our deformation-based approach has additional benefits that set it apart from existing modalities. For example, the addition of our proposed soft sensing approach into existing wearable paradigms could allow for monitoring a combination of external information about the muscle, such as deformation, with internal physiological parameters, such as electrical activity or lateral oscillations, providing the necessary data for powerful new research. For example, a combination of sEMG and the soft sensors could be used to estimate physiological electromechanical delay (EMD), which can be an indicative metric of impaired motor control or pain [43], [44]. EMD is a measure of the delay between the onset of electrical muscle excitation (captured by sEMG) and force production resulting in kinematic changes at the joint (captured by the soft sensors). In general, employing more than one sensing method has led to increased accuracy when compared to single sensing methodologies for a variety of applications, such as estimating muscle force, classifying activity, distinguishing between grasp types, or segmenting gait phases [12], [41], [45]–[47].

Although the soft sensors can detect small muscle deformations during a contraction, their sensitivity also induces high variability to placement during donning. This is due in large part to complex muscular anatomy within, and anatomical variability across, individuals. *Experiment 3* showed that the correlation between sensor and torque responses can change significantly when the sensors are placed out of a region of monotonic deformation during the full range of force production. For this reason, in this study we were careful to minimize any movement of the textiles relative to the underlying musculature. However, sensitivity to placement is also a known limitation in other wearable sensing modalities, and it is often addressed by increasing the density of sensors in the region of interest [40], [48].

While the use of an array with multiple sensors could facilitate the sensor placement process, it introduces the challenge of how to select the most suitable set of sensors for a given exercise. To select these sensors, we need to be able to determine which sensors on the array best correlate with force during a contraction without having a ground truth measure (e.g. the dynamometer). The small variability in the sensor-torque relationship between participants demonstrated in *experiment 1* Fig. 4B indicates similar changes in muscle deformation during a specific exercise, which could be useful in the sensor selection process. For example, in unconstrained environments, sensors could be selected by comparing the sensor responses during warm-up sets of a given exercise to the sensor responses of different participants performing the same exercise. The sensor profiles that are most similar to the average response from other participants would be selected, similar to the global fit identified for quadriceps deformation on a dynamometer. Moving forward, exploring improved initial sensor placement and more efficient calibration algorithms for selection will be needed to solve these problems.

True adoption of this approach in unconstrained environments will require further investigation and research to continue understanding the unknowns of deformation-based sensing with soft strain sensors. For example, this work only examines the deformation-torque relationship during muscle contractions, but does not analyze data during muscle relaxation. How muscle deformation differs in these two regimes remains unknown. Additionally, understanding and characterizing how the mechanical properties and nonlinearities of the chosen sensor technology and adhesive layer could help better quantify the sensor-measured deformation. Similarly, how skeletal muscle architecture (parallel, pennate, etc.) affects the fundamental relationship between force-generation, measured deformation, and joint torque will be important to understand. These questions guide exciting new research directions that will contribute to the advancement of using soft strain sensors for muscle activity monitoring.

Moving forward, we plan to use the soft sensors to monitor muscle activity during other types of exercises, such as cycling or bodyweight movements. We also aim to explore how these sensors could be used to monitor force development or track force reduction in other muscle groups. Ultimately, future work using these soft sensors in unconstrained environments will be necessary to explore the full potential of a continuous muscle force monitoring system based on muscle deformation.
